# Dose-Effectiveness Relationships Determining the Efficacy of Ibandronate for Management of Osteoporosis: A Meta-Analysis

**DOI:** 10.1097/MD.0000000000001007

**Published:** 2015-07-02

**Authors:** Yanjie Hou, Ke Gu, Chao Xu, Huiyong Ding, Changxin Liu, Yilihamu Tuoheti

**Affiliations:** From the Department of Orthopaedics (YH, CX, HD, YT), The Second Affiliated Hospital of Xinjiang Medical University, Urumchi; Department of Pain and Minimally Invasive (KG), The 316th Hospital of People's Liberation Army, Beijing; and Pain Center (CL), The First Affiliated Hospital of Beijing University of Chinese Medicine, Beijing, China.

## Abstract

The purpose of this study was to perform a meta-analysis on the efficacy of ibandronate by evaluating the effect sizes of different dosing regimens.

Major electronic databases were searched from 1985 to February 2015. A random effects meta-analysis was performed in STATA.

Data from 34 studies (13,639 patients) were included in this meta-analysis. Ibandronate treatment significantly improved lumbar spine bone mineral density (BMD) as shown by the percent change from baseline (4.80%, *P* < 0.0001, 95% confidence interval [CI] [4.14, 5.45]). The respective effect sizes for oral intake and intravenous (IV) infusion were 4.57% and 5.22% (*P* < 0.0001, CIs [3.71, 5.42] and [4.37, 6.07]), respectively. All doses led to a significant increase in BMD except 2 oral dose regimens (1 mg/d: 4.65%, *P* = 0.285, 95% CI [−3.87, 13.18] and 0.5 mg/d: 3.60%, *P* = 0.38, 95% CI [−4.43, 11.64]. Ibandronate treatment (overall as well as dose wise) also significantly improved the total hip BMD—2.30% overall, 2.13% oral, and 2.63% IV (*P* < 0.0001, 95% CIs [1.96, 2.64], [1.70, 2.55], and [2.07, 3.20]), respectively. Ibandronate administration significantly decreased serum markers of bone resorption to −46.53% for C-terminal telopeptide of type 1 collagen, −24.03% for bone-specific alkaline phosphatase, and −50.17% for procollagen type I N-terminal propeptide (*P* < 0.0001, 95% CIs [−53.16, −39.91], [−31.28, −16.77], and [−64.13, −36.20]), respectively. Parathyroid hormone levels remained unaffected by ibandronate treatment (3.03%, *P* = 0.439, 95% CI [−5.06, 11.66]).

There was no significant difference in the efficacy of ibandronate between oral or IV administration. Predominant dose regimens for IV administration were 1 to 3 mg/3 mo and 150 mg/mo oral and 2.5 mg/d for oral ibandronate treatment.

## Abstract 2

Supplemental Digital Content is available in the text

## INTRODUCTION

Osteoporosis is a state of bone fragility that increases susceptibility of the patients to fractures. It is an important global public health concern with both societal and economic implications. About 75 million people in the United States, Europe, and Japan suffer from osteoporosis.^1^ It is estimated that the incidence of hip fracture will increase by up to 240% in women and 310% in men by the year 2050.^2^

Osteoporosis is strongly associated with age and causes significant morbidity and mortality in the elderly, affecting both men and women. Although osteoporotic fractures can occur anywhere in the skeletal system, vertebral, hip, and wrist fractures are most common. Vertebral fractures may cause height loss and respiratory dysfunction, which subsequently leads to reduced quality of life, social withdrawal, and morbidity.^3,4^ Hip fractures are associated with significantly increased mortality rates, with most mortality events occurring within 3 to 6 months after the event.^5^ The lifetime risk of fracture incidence is higher in women. Ten-year fracture risk at 50 years of age is 9.8% in women and 7.1% in men, which increases to 21.7% and 8%, respectively, by 80 years of age.^6^

Therapeutic options for the management of osteoporotic fractures include the use of bisphosphonates, parathyroid hormone (PTH) analogs, selective estrogen receptor modulators, denosumab (antireceptor activator of nuclear factor-κ B ligand antibody), tibolone, calcitonin, and strontium ranelate.^7^ Among these, bisphosphonates, which are most commonly used, reduce osteoclast-mediated bone resorption.^8^ Although, nonnitrogenous bisphosphonates such as clodronate and etidronate are also used, nitrogenous bisphosphonates such as alendronate, ibandronate, risedronate, and zoledronate are more efficacious.^5^

An inherent constraint in the use of bisphosphonates is their poor bioaccessibility. Oral intake leads to <1% absorption in the gut. Moreover, fasting prior to administration is required and the patient must not lie down for 30 minutes following administration because these drugs cause esophageal irritation.^9^ This has led to poor patient compliance and compromised treatment efficacy. Alternatively, bisphosphonates can be infused via the intravenous (IV) route, which greatly enhances their bioavailability and reduces the frequency of administration compared to oral intake.

Despite the superior efficacy of IV infusions, oral intake of bisphosphonates remains common. Ibandronate is a preferable option within the bisphosphonate group as it offers relatively flexible dosing formulations and intake schedules. A number of trials have attempted to examine the efficacy of either oral intake or IV infusion of the bisphosphonates; others have compared both regimens. However, there is a paucity of trials with placebo-controlled designs. The objective of this study was to carry out a random effects meta-analysis, pooling data from trials focusing on the dose-effectiveness relationships of ibandronate therapy and the efficacy of oral versus IV administration.

## METHOD

### Ethical Review

Meta-analysis does not involve ethical review.

### Literature Search

Several electronic databases including EBSCO, Embase, Google Scholar, Ovid SP, PubMed, Scopus, and Web of Science were used for the literature search. The major medical subject headings and keywords—bisphosphonates, nitrogen containing bisphosphonates, ibandronate, osteoporosis, fracture, bone mineral density (BMD), calcium, phosphate, lumbar spine, vertebral, hip, osteocalcin, sclerostin, C-terminal telopeptide of type 1 collagen (CTX), bone-specific alkaline phosphatase (BSAP), procollagen type I N-terminal propeptide (PINP), PTH, vitamin D, clinical trial, oral, IV, etc—were used in different logical combinations and phrases. The search encompassed original research articles published from1985 to February 2015.

### Inclusion and Exclusion Criteria

The inclusion criteria were trials recruiting osteoporosis patients or vulnerable populations to study the efficacy and safety of the ibandronate for at least 1 year period; where BMD of lumbar spine and/or total hip was measured; and providing baseline and final values or percent change from baseline. Exclusion criteria were trials utilizing ibandronate for purposes other than skeletal improvement; to assess patient adherence only; in combination with other therapeutic regimens such as PTH; to study the safety profile only; and with relevant but inadequate information for the meta-analysis.

### Data Extraction, Synthesis, and Statistical Analyses

Important information including outcome measures and outcomes, primary and secondary endpoints, dosage and mode of administration, serum markers of osteoporosis development/improvement, BMD, and participants’ demographic characteristics were extracted onto datasheets. The meta-analysis was carried out by using Stata software (Version 12; StataCorp, College Station, TX). The random effects model was used, pooling the means and standard deviations of the variables of interest from all relevant studies. The effect sizes of subgroups were then subjected to a z test in order to evaluate the significance of difference. Statistical heterogeneity between the studies was tested by *I*^2^ index. Sensitivity analyses were performed, wherever necessary. Egger and Begg tests were performed to examine the publication bias.

## RESULTS

**FIGURE 1:**
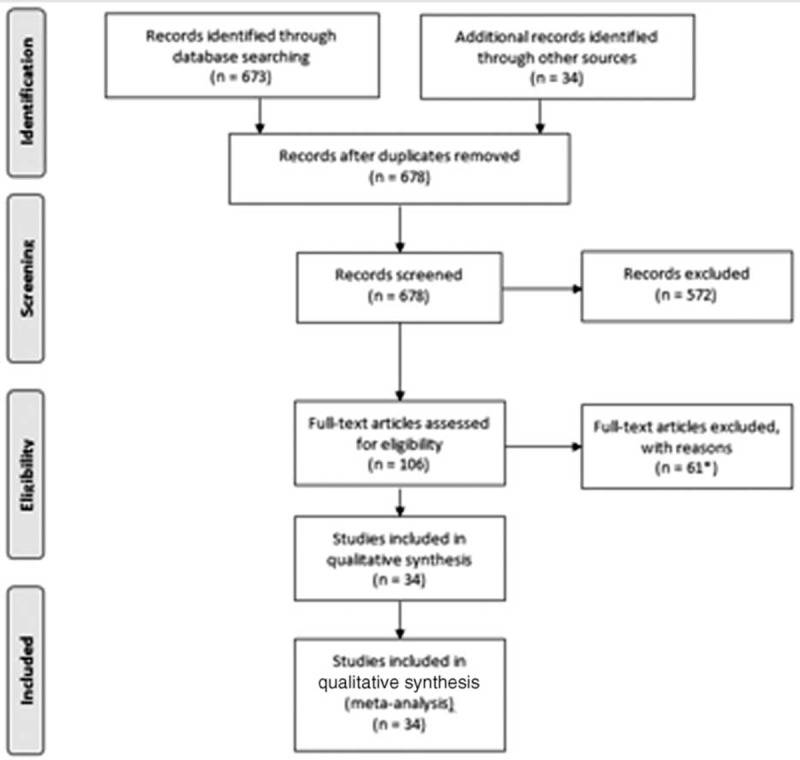
Flowchart of literature search, study screening, and selection process. ^∗^Results of 34 studies were published in 45 articles.

Thirty-four studies fulfilled the eligibility criteria. Results of these trials were published in 45 articles^10–54^ and a flowchart summarizing study screening and the selection process is presented in Figure 1. Briefly, the following studies were included in the meta-analysis: 28 randomized controlled, 1 nonrandomized controlled,^11^ 4 prospective observational,^22,26,32,53^ and 1 retrospective^46^ study. Of the studies included, their important characteristics are presented in Table S1, http://links.lww.com/MD/A306. Publication bias tests indicated the chances of significant bias (Table S2, http://links.lww.com/MD/A306; Figures S1a and b, http://links.lww.com/MD/A306).

Overall, 11,090 patients received ibandronate, whereas 2549 patients were used as placebo controls. Among the ibandronate-treated patients, 7531 were administered oral ibandronate and 3559 received it as IV infusions. Among the important demographic data, age, height, weight, and body mass index as mean and standard deviation were 62.44 ± 7.57 years, 159.07 ± 6.68 cm, 64.56 ± 12.4 kg, and 25.34 ± 4.38 kg/m^2^, respectively.

Average duration of ibandronate treatment in these trials was 1.9 ± 1.06 (1–5) years. Prior to entering the trial, 45.7% of the patients had a history of fractures. Average time since menopause in women with postmenopausal osteoporosis (PMO) was 15.39 ± 7.04 years. At the time of entry into the trial, these participants had serum markers measured as vitamin D (30.21 ± 11.6 ng/mL), PTH (49.9 ± 25.24 pg/mL), osteocalcin (23.65 ± 9.88 ng/mL), serum CTX (0.42 ± 0.29 ng/mL), serum PINP (49.73 ± 31.16 ng/mL), BSAP (58.75 ± 19.71 U/L), serum calcium (9.45 ± 0.519 mg/dL), and serum phosphate (3.66 ± 0.588 mg/dL).

**FIGURE 2:**
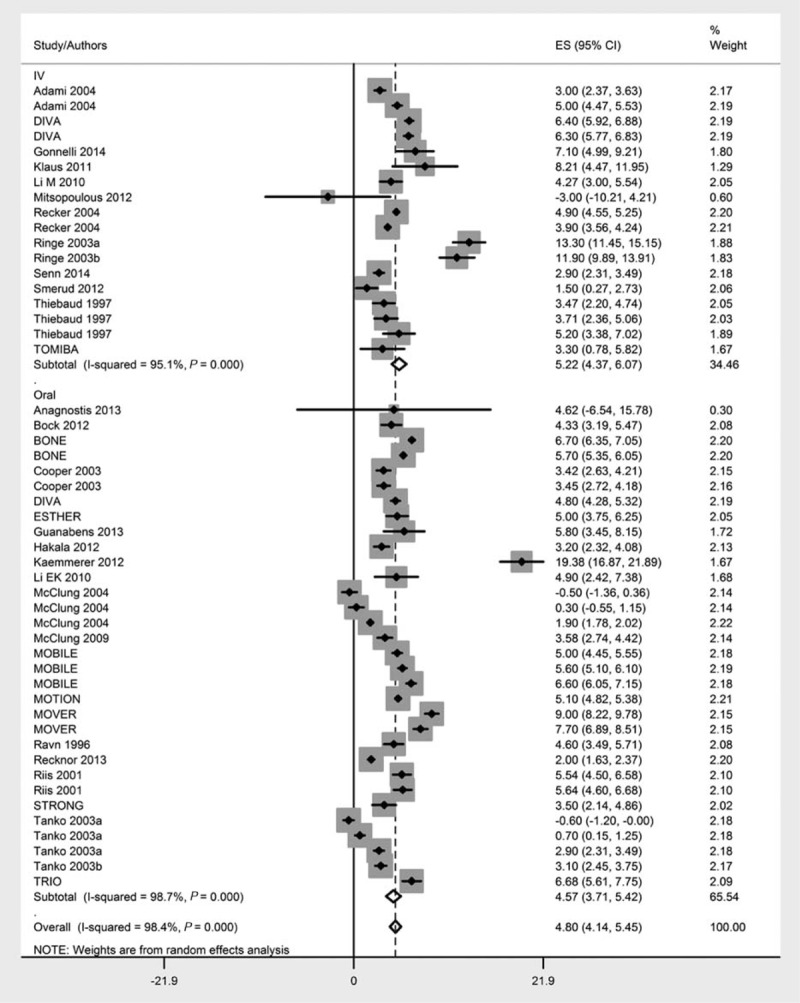
Forest chart showing the effect sizes of individual studies and overall effect sizes with differentiation of intravenous and oral administration achieved in this meta-analysis. Effect sizes represent percent change in the bone mineral density following ibandronate treatment.

Ibandronate treatment significantly improved lumbar spine BMD. The overall effect size (percent change from baseline) was 4.80%, *P* < 0.0001, 95% CI [4.14, 5.45]. Oral intake of ibandronate led to a change of 4.57%, *P* < 0.0001, 95% CI [3.71, 5.42], whereas the effect size of IV infusion was 5.22%, *P* < 0.0001, 95% CI [4.37, 6.07] (Figure 2). There was no significant difference between the efficacy of oral and IV ibandronate administration (z = 0.264; *P* = 0.791).

**FIGURE 3:**
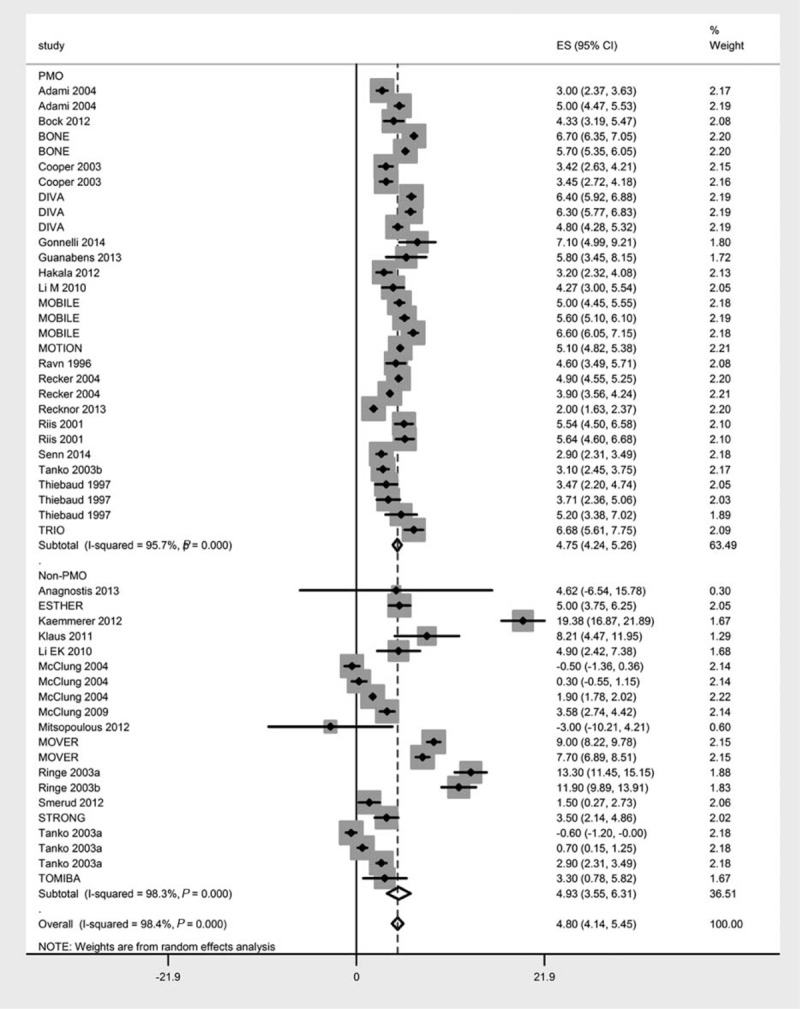
Forest chart showing the effect sizes (percent change in the bone mineral density following ibandronate treatment) of postmenopausal women versus all other osteoporotic conditions.

A subgroup analysis to examine the difference of ibandronate efficacy in improving lumbar spine BMD in postmenopausal women versus all other osteoporotic conditions revealed no significant difference (4.75% vs 4.93%, 95% CIs [4.24, 5.26] and [3.55, 6.31]) between subgroups (z = 0.067; *P* = 0.95; Figure 3). Similarly, there was no significant difference in the percent change from baseline in the lumbar spine BMD between males (5.96% [2.92, 8.99]) and females (4.547% [3.88, 5.21, 4.75]) between subgroups (z = 0.90; *P* = 0.367; Figure S2, http://links.lww.com/MD/A306).

**FIGURE 4:**
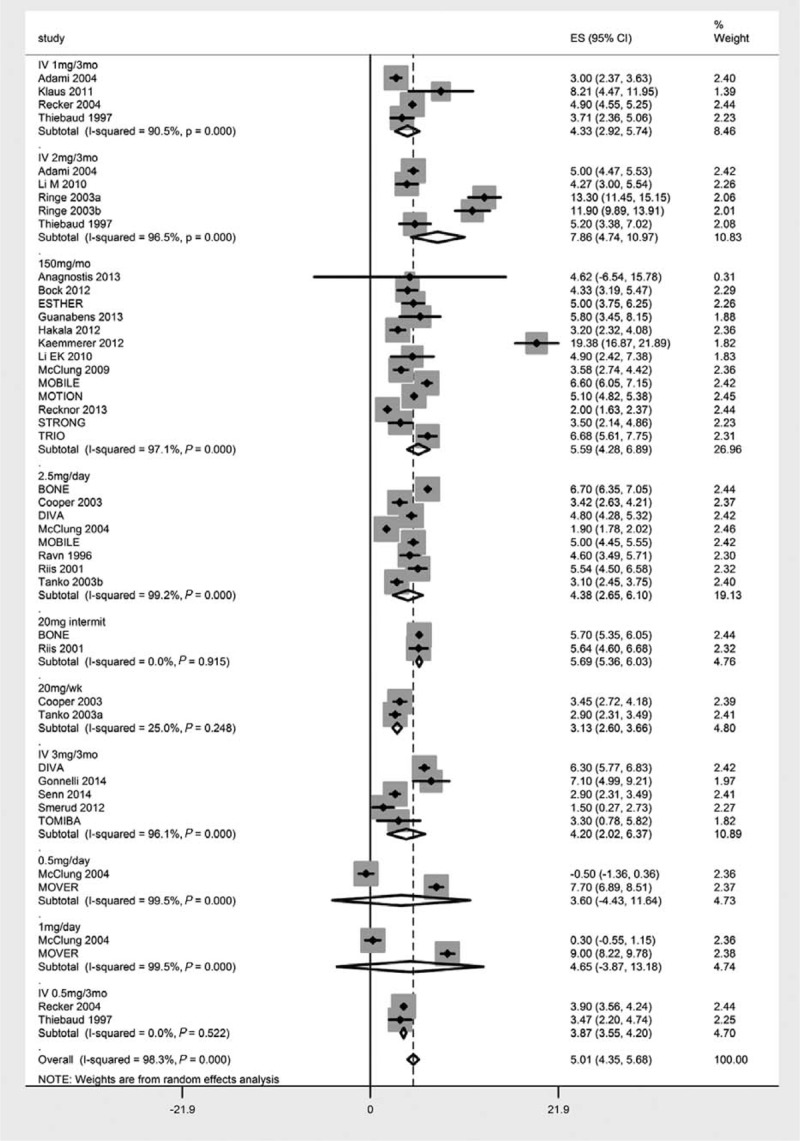
Forest chart showing dose-wise effect sizes (percent change in the bone mineral density following ibandronate treatment) achieved in this meta-analysis.

The effect sizes (percent changes in the BMD of lumbar spine) of different doses of orally administered and intravenously infused ibandronate are presented in Table 1 and Figure 4. Only 2 oral dose regimens led to nonsignificant increase in lumbar spine BMD (1 mg/d: 4.65%, *P* = 0.285, 95% CI [−3.87, 13.18] and 0.5 mg/d: 3.60%, *P* = 0.38, 95% CI [−4.43, 11.64]). In the between-dose subgroup analyses, the efficacy of IV 2 mg/3 mo differed significantly from IV 0.5 mg/3 mo (z = 2.5; *P* = 0.0124) and the efficacy of oral 150 mg/mo differed significantly from oral 0.5 mg/d (z = 0.479; *P* = 0.632). None of the other dose regimens differed significantly in affecting lumbar spine BMD. Besides this, one study each also could not find any significant change in BMD from IV 1 mg/mo,^32^ oral 5 and 10 mg/wk doses.^50^

**TABLE 1:**
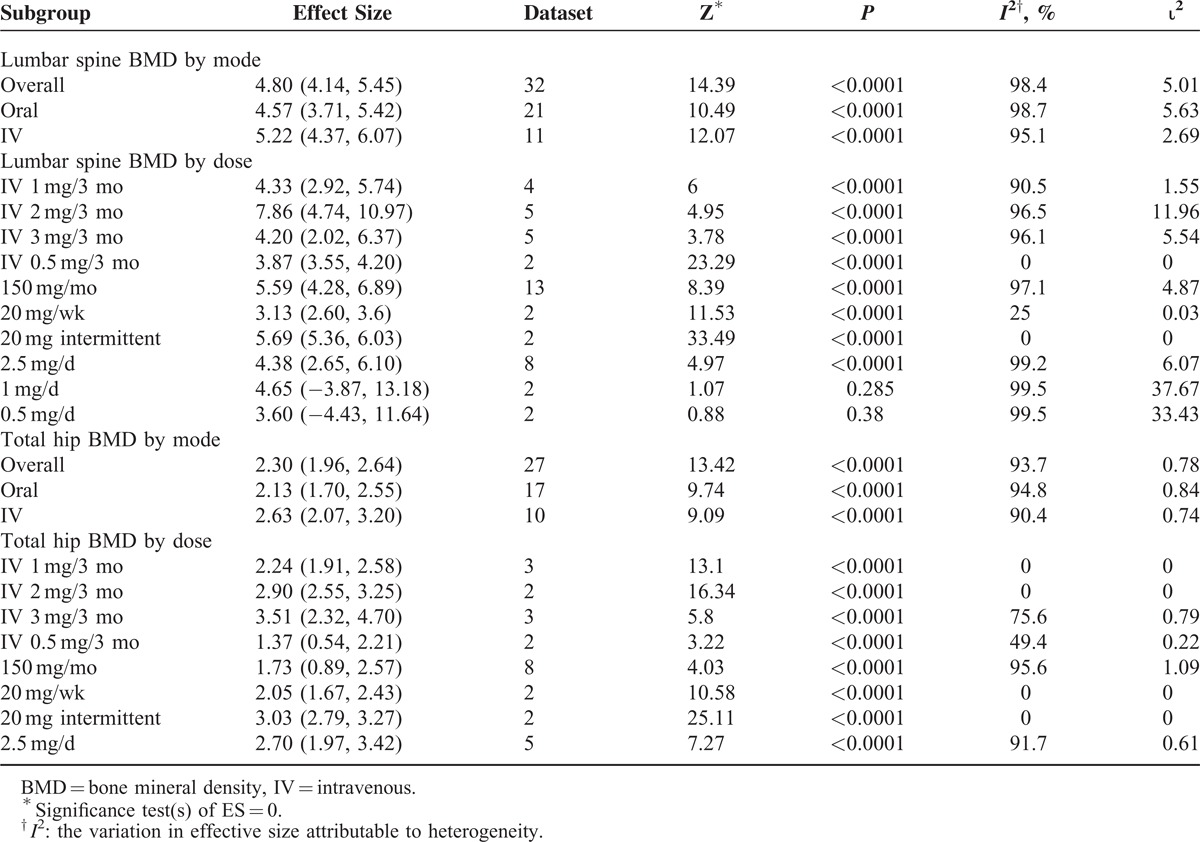
Overall, by Mode of Administration and Dose Regimen Meta-Analyses, Outcomes (Percent Changes From Baseline in the BMD After Ibandronate Treatment)

Ibandronate treatment also improved total hip BMD significantly. The overall effect size (percent change from baseline) was 2.30%, *P* < 0.0001, 95% CI [1.96, 2.64]. Oral intake of ibandronate led to a change of 2.13%, *P* < 0.0001, 95% CI [1.70, 2.55], whereas the effect size of IV infusion mode was 2.63%, *P* < 0.0001, 95% CI [2.07, 3.20] (Figure S3, http://links.lww.com/MD/A306). There was no significant difference between the efficacy of these 2 routes of ibandronate administration (z = 1.389; *P* = 0.1645). The effect sizes of different doses of orally administered and intravenously infused ibandronate are presented in Table 1 and Figure S4, http://links.lww.com/MD/A306. None of the dose regimens differed significantly in affecting total hip BMD.

Both the modes of ibandronate administration significantly decreased serum markers of bone resorption (Table 2). Percent changes from baseline in these markers were −46.53%, *P* < 0.000, 95% CI [−53.16, −39.91] for CTX, −24.03%, *P* < 0.0001, 95% CI [−31.28, −16.77] for BSAP, and −50.17%, *P* < 0.0001, 95% CI [−64.13, −36.20] for PINP. There were no significant differences in the changes in these serum markers with regard to the mode of administration. Parathyroid hormone levels remained unaffected from ibandronate treatment (3.03%, *P* = 0.439, 95% CI [−5.06, 11.66]).

**TABLE 2:**
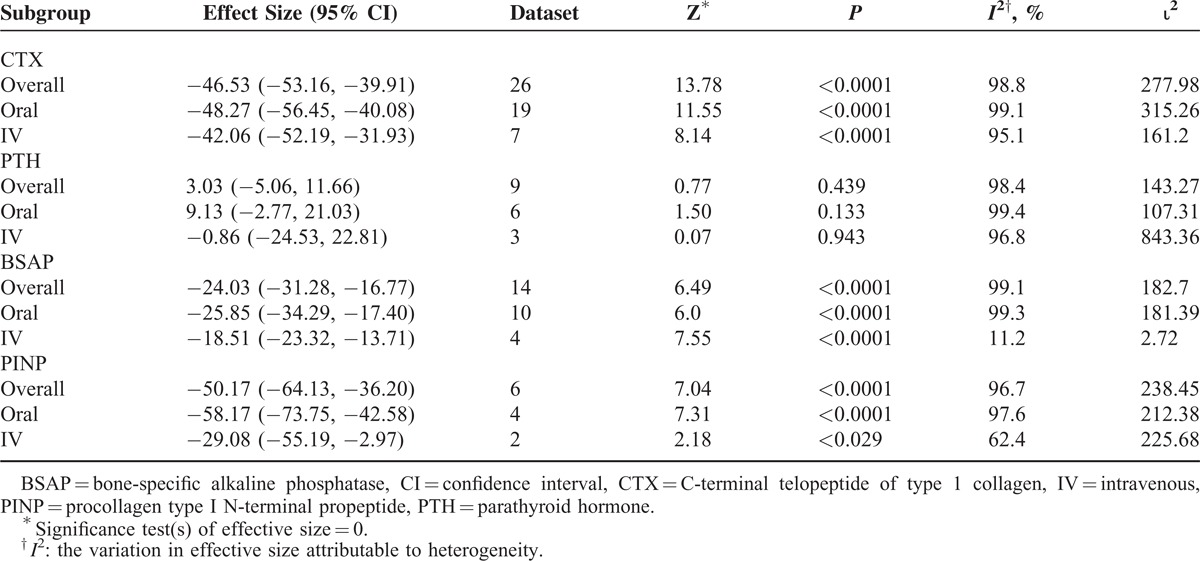
Percent Changes From the Baseline in the Serum Markers After Ibandronate Treatment

## DISCUSSION

This study was designed to seek updated evidence regarding the dose-wise efficacy of ibandronate in the treatment or prevention of osteoporosis. The majority of dose regimens were found to be significantly efficacious and there was no significant difference between the efficacies of IV ibandronate infusions of 1 to 3 mg every third month or orally administered doses including 150 mg/mo, 20 mg/wk, or 1 to 2.5 mg daily. Only 0.5 and 1 mg/d oral dose regimens led to nonsignificant increases in lumbar spine BMD.

Within the ibandronate treatment period (about 2 years, on average), the annual incidence of fractures was 2.34 ± 1.58% in the study population in which 45.73 ± 23.41% patients had a history of fractures. Data from placebo-controlled studies included in this meta-analysis revealed that annual incidence of fractures during the treatment period was 3.52 ± 2.31% in placebo versus 2.1 ± 1.02% in ibandronate groups when the percent increase in the BMD was 4.22% in lumbar spine and 2.15% in total hip in the ibandronate-treated participants of these placebo-controlled trials. Thus, ibandronate treatment was associated with a 1.42 ± 2.52% reduction in the annual incidence of fractures. These results further support the notion that BMD is a strong predictor of fracture risk and is, therefore, an appropriate surrogate marker of bone strength.^55^

So far, it is known that ibandronate therapy reduces vertebral fracture risk, but evidence is inconclusive for nonvertebral fracture as well as hip fracture risk reduction. In general, in comparison with oral 2.5 mg/d dose, oral 150 mg/mo or IV ibandronate treatments are associated with a longer time to fracture event and lower fracture rates.^56^

In this study, we have noted a slightly higher ibandronate efficacy in males than females, but this finding was not statistically significant. In females, estrogen status is an important determinant of bone health as has been demonstrated in an ovariectomized primate model.^57^ A relatively higher risk of osteoporotic fractures in women is also attributed to the anatomical differences. Although trabecular thinning with increasing age is seen in both the sexes, trabecular dropout is observed only in women. Men have larger bones with a lesser degree of cortical thinning with age.^58^ However, although the risk is lower, osteoporotic fractures constitute an important cause of morbidity and mortality also in men.^59^

Timely treatment initiation and adherence to ibandronate therapy can increase efficacious outcomes. Intravenous administration of ibandronate prevents gastrointestinal intolerance and ensures better compliance leading to improved efficacy. However, tolerability characteristics such as the acute phase (flu-like) cytokine response and safety properties such as the risk of oversuppressed bone turnover, renal toxicity, and jaw osteonecrosis impose concerns over IV use.^60,61^ On the contrary, the complex dosing modalities of oral route administration including fasting, regularity, and adverse effects can compromise compliance and adherence to regular intake.^62^

With its multioption dosing, convenient IV infusion, and better safety profile, ibandronate appears to have advantageous over its contemporaneous oral or IV bisphosphonates. In a pooled analysis of clinical trials with over 6000 subjects, ibandronate treatment was not found to increase the risk of atrial fibrillation.^63^ The bioavailability of ibandronate also varies in different geographic populations. Although the bioavailability of oral ibandronate is 0.91% in a Japanese population, it is 0.63% in western populations. Thus, an optimal oral dose of 100 mg/mo ibandronate is suggested in Japan, but 150 mg/mo in the west.^64^ This factor might also have some impact on the outcomes of this meta-analysis.

## CONCLUSION

Both routes of ibandronate administration significantly increase BMD and thus potentially reduce the risk of osteoporotic fractures. Overall change in BMD following ibandronate treatment did not differ significantly by oral versus IV administration or by PMO versus other forms of osteoporosis or sex. Only low doses of oral administration (0.5 and 1 mg/d) produce a nonsignificant increase in BMD. Serum markers of bone resorption including BSAP, CTX, and PINP are significantly reduced in the ibandronate-treated patients. Parathyroid hormone levels remained unaffected by the ibandronate treatment.
